# Effect and Mechanism of Herbal Medicines on Cisplatin-Induced Anorexia

**DOI:** 10.3390/ph15020208

**Published:** 2022-02-09

**Authors:** Daeun Min, Bonglee Kim, Seong-Gyu Ko, Woojin Kim

**Affiliations:** 1 Department of Physiology, College of Korean Medicine, Kyung Hee University, Seoul 02453, Korea; wndqhr1456@gmail.com; 2 Korean Medicine-Based Drug Repositioning Cancer Research Center, College of Korean Medicine, Kyung Hee University, Seoul 022447, Korea; bongleekim@khu.ac.kr (B.K.); epiko@khu.ac.kr (S.-G.K.)

**Keywords:** anorexia, chemotherapy-induced side effects, cisplatin, herbal medicines

## Abstract

Cisplatin is a well-known chemotherapeutic agent used to treat various types of cancers; however, it can also induce anorexia, which results in reduced food intake, loss of body weight, and lower quality of life. Although drugs such as megestrol acetate and cyproheptadine are used to decrease this severe feeding disorder, they can also induce side effects, such as diarrhea and somnolence, which limit their widespread use. Various types of herbal medicines have long been used to prevent and treat numerous gastrointestinal tract diseases; however, to date, no study has been conducted to analyze and summarize their effects on cisplatin-induced anorexia. In this paper, we analyze 12 animal studies that used either a single herbal medicine extract or mixtures thereof to decrease cisplatin-induced anorexia. Among the herbal medicines, Ginseng Radix was the most used, as it was included in seven studies, whereas both Glycyrrhizae Radix et Rhizoma and Angelicae Gigantis Radix were used in four studies. As for the mechanisms of action, the roles of serotonin and its receptors, cytokines, white blood cells, ghrelin, and leptin were investigated. Based on these results, we suggest that herbal medicines could be considered a useful treatment method for cisplatin-induced anorexia.

## 1. Introduction

Cisplatin (also known as cisplatinum or *cis*-diamminedichloroplatinum (II)), is a first-generation platinum-based chemotherapeutic agent widely used to treat solid cancers, such as testicular, lung, ovarian, and breast cancer, since its approval by the Food and Drug Administration (FDA) in 1978 [1,2,3]. However, along with its remarkable antitumor effect, cisplatin can also induce several side effects, such as nephrotoxicity, allergic reactions, decreased immunity to infections, and diverse gastrointestinal (GI) disorders [4,5,6]. In particular, GI disorders such as diarrhea, nausea, vomiting, and anorexia are clinically important, as they can lead to the discontinuation of therapy due to reduced dietary intake and abnormal metabolism [7,8,9,10,11]. Among them, anorexia is particularly important, as it has been reported that approximately half of patients with cancer suffer from anorexia after chemotherapy treatments [12,13,14,15,16].

Anorexia is a loss of appetite, including the occurrence of early satiety, which results in reduced food intake, loss of body weight, and lower quality of life [17]. Furthermore, when these symptoms become chronic, a loss of fat and muscle occurs, which often leads to cachexia [18,19,20]. Megestrol acetate (MGA) [21,22,23,24] and cyproheptadine [25,26] are used as first-line treatments to attenuate anorexia. They have been reported to enhance appetite and increase the body weight of patients with chemotherapy-induced anorexia [27,28,29]. However, both MGA and cyproheptadine are known to cause adverse effects. MGA is associated with impotence, deep-vein thrombosis, and gastrointestinal complications, such as diarrhea [30,31], and cyproheptadine has been reported to cause somnolence, hallucinations, tachycardia, and muscle twitching [32,33]. Therefore, efforts to find new treatments that could attenuate anorexia with fewer side effects are needed.

Herbal medicines have been used as a relatively safe remedy for many years to prevent and treat numerous diseases, making it possible to promote health and enhance the quality of life [34,35]. In particular, they have been shown to be effective against diverse GI tract disorders, such as dyspepsia, constipation, nausea, and vomiting [36,37,38]. In clinical settings, ban-xia-xie-xin-tang, a classical herbal mixture from one of the earliest books of traditional medicine, was shown to be effective in patients with chemotherapy-induced diarrhea through a significant improvement and reduction in the frequency of diarrhea with higher grades [39,40]. Another well-known herbal medicine, Dai-kenchu-to, increased the duodenum and jejunum motility in dogs when administered intraduodenally and intrajejunally [41]. Moreover, isolated guinea pig ileum Dai-kenchu-to was also shown to induce contractions accompanied by autonomous contractions [42].

In our lab, we have previously shown that orally administered herbal mixtures, such as Sip-Jeon-Dae-Bo-Tang (SJDBT) [43] and LA16001 [44], could effectively attenuate cisplatin-induced appetite loss in mice. These results suggest that herbal medicines could be considered a useful option to attenuate cisplatin-induced anorexia. Although dozens of studies have been published in the last 20 years to demonstrate the effect of herbal medicines on cisplatin-induced anorexia, to date, no reviews summarizing and analyzing their effects on cisplatin-induced feeding disorders have been published. Thus, by analyzing 12 studies that used either a single herbal medicine extract or mixtures thereof to treat cisplatin-induced anorexia in rodents, their therapeutic effect and the underlying mechanism of action are discussed.

## 2. Results

The included studies focused on various pathways as the pathogenesis mechanism of cisplatin-induced anorexia, as well as the curative action of herbal medicines (Table 1 and Table 2). The role of 5-HT and its receptors in cisplatin-induced anorexia was investigated in four studies [45,46,47,48]; inflammatory cytokines [43,44,47,49] and WBCs [44,47,49,50] were also studied in four studies each; the roles of ghrelin and leptin in cisplatin-induced anorexia were assessed in five [43,44,45,46,48] and two [43,44] studies, respectively. 

### 2.1. 5-HT and Its Receptors

Serotonin (5-HT) and its receptors are involved in several physiological and pathological functions of the GI tract, including motility, secretion, and weight maintenance [49]. In the brain, 5-HT is synthesized in the dorsal and median raphe and innervates most of brain regions related to feeding regulation, including the hypothalamus, hippocampus, amygdala, and frontal cortex [50]. In the periphery, 5-HT is mostly secreted in the GI tract, especially in the secretory granules of enterochromaffin cells [51]. GI disorders are known to occur when large amounts of 5-HT are released from enterochromaffin cells and bind to 5-HT receptors [45]. Loss of appetite has also been shown to be closely related to 5-HT and its receptors, as food intake is significantly reduced following intraperitoneal injection of 5-HT [52,53,54]. In this review, four studies observed the role of 5-HT and its receptors in cisplatin-induced anorexia [45,46,47,48]. 

In a study by Takeda et al. [45], a single intraperitoneal injection of cisplatin induced a decrease in 6 h food intake in rats; however, the administration of 5-HT_2B_ or 5-HT_2C_ receptor antagonists (i.p.) attenuated the decrease in food intake, indicating that the activation of 5-HT receptors plays an important role in the development of cisplatin-induced anorexia. The authors further assessed that the plasma-acylated ghrelin level, which is the activated form of ghrelin, was significantly decreased in rats treated with both cisplatin and 5-HT_2B_ or 5-HT_2C_ receptor agonists. However, injection of 5-HT_3_ or 5-HT_4_ receptor agonists failed to alter the acylated ghrelin level. In this study, rikkunshito (RKT), which is composed of eight herbal medicines, improved cisplatin-induced anorexia. Among the crude drug components of RKT, 3,3′,4′,5,6,7,8-heptamethoxyflavone (HMF), hesperetin and isoliquiritigenin (found in Aurantii nobilis Pericarpium), Glycyrrhizae Radix et Rhizoma, and Zingiberis Rhizoma, showed significant inhibitory constant (*Ki*) values against the 5-HT_2B_ receptor. Moreover, hesperetin and isoliquiritigenin also showed significant *Ki* values against the 5-HT_2C_ receptor, suggesting that inhibiting the action of 5-HT_2B_ or 5-HT_2C_ receptors may prevent a decrease in food intake induced by cisplatin injection. In accordance with the study of Takeda et al., Yakabi et al. [46] also found that 5-HT_2C_ receptors are involved in cisplatin-induced anorexia, as a 5-HT_2C_ receptor antagonist (SB242084HCL) treatment reversed the decrease in appetite induced by cisplatin, whereas 5-HT_3_ receptor antagonists (granisetron or ondansetron) did not. Altogether, these results suggest that 5-HT_2B_ and 5-HT_2C_ receptors, but not 5-HT_3_ and 5-HT_4_ receptors, are involved in cisplatin-induced anorexia. 

The role of 5-HT in the small intestine was observed by Kim et al. [47]. They showed that 5-HT concentration in small-intestine tissue increased by 1.8-fold compared to that in the normal group after cisplatin injection. In their study, water extract of Rhus verniciflua Stoke (RVX) attenuated anorexia and succeeded in decreasing 5-HT levels in the small intestine, indicating that modulating 5-HT in the small intestine may be an effective treatment for anorexia. Furthermore, RVX significantly upregulated serotonin transporters (SERT) and downregulated 5-HT_3A_ receptors in the small intestine. SERT transports 5-HT to the epithelial cells of the GI tract, where 5-HT is metabolized. It has been reported that cisplatin injection decreases the number of SERT [55]. Thus, damage to epithelial cells due to cisplatin can increase the action of 5-HT to its receptors, such as 5-HT_3A_, whose activation is known to be related to chemotherapy-induced emesis [56]. 

In a study by Song et al. [48], He-Wei granules (HWKL), which are formulated from seven herbs, increased 5-hydroxyindole acetic-acid (5-HIAA) content and lowered 5-HT and substance P levels, which had been elevated following cisplatin injection, thus increasing the ratio of 5-HIAA/5-HT in the serum, medulla oblongata, and ileum part of the small intestine. The 5-HIAA/5-HT ratio is used to represent the effect of a drug on 5-HT [57,58], and an increase in the ratio can be interpreted as a decreased effect of 5-HT due to its metabolism to 5-HIAA. In addition, Song et al. confirmed that HWKL significantly reduced 5-HT_3A_ receptor mRNA, and protein-expression levels in both the ileum and medulla oblongata increased after cisplatin treatment. Furthermore, the upregulated substance-P levels in the serum, medulla oblongata, and ileum decreased after HWKL administration. Substance P is a neurotransmitter produced from enterochromaffin cells similar to 5-HT that is involved in feeding behavior, and its intraperitoneal injection has been shown to reduce food intake [59,60]. Moreover, it is reported that substance P is upregulated via the interaction between 5-HT, which is increased by cisplatin injection, and the 5-HT_3_ receptor [61,62]. 

### 2.2. Inflammatory Cytokines

Compelling studies have demonstrated that anorexia is associated with inflammation [63]. Injection of proinflammatory cytokines results in reduced food intake in rodents [64]. In patients with anorexia nervosa, an increase in pro-inflammatory cytokines, such as interleukin-1β (IL-1β), tumor necrosis factor-α (TNF-α), and interleukin-6 (IL-6), have also been reported [65]. IL-1β has been reported to suppress feeding by reducing meal frequency and size through several pathways, including both neural and humoral pathways [63,66,67,68]. TNF-α [69,70,71] and IL-6 [72] have also been reported to influence feeding behavior [73,74]. In this review, the relationship between changes in inflammatory cytokines in cisplatin-induced anorexia was analyzed in four studies [43,44,47,49].

**Table 1:** The effect of herbal medicine extracts in cisplatin-induced anorexia.

Authors/ Years	Animal Type	Cisplatin Dosing	Herbal Medicine/ Dose	Findings				
				Group	Food Intake	Kaolin Intake	Body Weight	Mechanisms of Actions
Aung et al. 2003 [75]	Wistar rats	Single 3 mg/kg, i.p.	Scutellaria baicalensis roots [SbE] (water extract) 1, 3, and 10 mg/kg, i.p.	Cisplatin (n = 7)	↓	↑	-	-
				Cisplatin + SbE (n = 6)	↑	↓	-	-
Mehendale et al. 2005 [76]	Wistar rats	Single 3 mg/kg, i.p.	American ginseng berries [AGBE] (75% ethanol extract) 50, 100, and 150 mg/kg, i.p.	Cisplatin (n = 6)	↓	↑	-	-
				Cisplatin + AGBE (n = 6)	↑	↓	-	-
				Cisplatin + Ginsenoside Re (5 mg/kg, i.p.) (n = 6)	-	↓	-	-
Takeda et al. 2008 [45]	SD rats	Single 2 mg/kg, i.p.	Rikkunshito [RKT]; Atractylodis Lanceae Rhizoma, Ginseng Radix, Pinelliae Tuber, Hoelen, Zizyphi Fructus, Aurantii Nobilis Pericarpium, Glycyrrhizae Radix, Zingiberis Rhizoma). (water extract) 500, 1000 mg/kg, p.o.	Cisplatin (n = 4–5)	↓	-	-	Plasma-acylated ghrelin↓ Plasma-desacylated ghrelin↓
				5-HT (4 & 8 mg/kg, i.p.) (n = 4–5)	-	-	-	Plasma-acylated ghrelin↓ Plasma-desacylated ghrelin↓
				Cisplatin + RKT (n = 4–5)	↑	-	-	Plasma-acylated ghrelin↑
				Cisplatin + RKT + GHS-R1a antagonist (0.4 μmol/rat, i.p.) (n = 4–5)	↓	-	-	-
				Cisplatin + HMF or Hesperetin or Isoliquiritigenin (n = 4–5)	-	-	-	Plasma-acylated ghrelin↑
				HMF, Hesperetin, Isoliquiritigenin (n = 4–5)	-	-	-	5-HT_2B_ receptor: Ki: 0.21 ± 0.01 & 5.3 ± 0.1 & 3.3 μmol/L
				Hesperetin, Isoliquiritigenin (n = 4–5)	-	-	-	5-HT_2C_ receptor: Ki: 20.9 ± 7.1 & 3.5 ± 0.1 μmol/L
Yakabi et al. 2010 [46]	SD rats	Single 2 mg/kg, i.p.	[RKT] (water extract) 500, 1000 mg/kg, p.o.	Cisplatin (n = 4–5)	↓	-	-	Hypothalamic GHS-R1a↓
				Cisplatin + Ghrelin (2 nmol/rat, I.C.V.) (n = 4–5)	↓	-	-	-
				5-HT_2C_ receptor agonist (9 mg/kg, i.p.) (n = 4–5)	↓	-	-	-
				5-HT_2C_ receptor agonist + RKT (n = 4–5)	↑	-	-	-
				Cisplatin + RKT (n = 4–5)	↑	-	-	Hypothalamic GHS-R1a↑ Effect abolished by GHS-R1a antagonist (1nmol/rat, I.C.V.)
				Cisplatin + Hesperidin or Isoliquiritigenin (n = 4–5)	↑	-	-	Effect abolished by GHS-R1a antagonist (1 nmol/rat, I.C.V.)
Raghavendran et al. 2011 [77]	SD rats	Single Pre—7 mg/kg Post—6 mg/kg, i.p.	Korean ginseng roots [KG; the root of Panax ginseng CA Meyer] (water extract) Pre—25, 50, 100 mg/kg Post—12.5, 25, 50 mg/kg p.o.	Cisplatin (n = 8)	↓	↑	-	Total WBC↑ Neutrophil↑ Lymphocyte↑ Stomach injury↑ Small intestine injury↑
				Cisplatin + KG (pre-treatment) (n = 8)	↑	↓	-	Total WBC↓ Neutrophil↓ Lymphocyte↓ Stomach injury↓ Small intestine injury↓
				Cisplatin + KG (post-treatment) (n = 8)	↑	↓	-	-
Woo et al. 2016 [43]	Balb/c mice	Single 8 mg/kg, i.p.	Sip-jeon-dea-bo-tang [SJDBT; Angelicae Gigantis Radix, Astragali Radix, Atractylodis Rhizoma Alba, Cinnamomi Cortex, Cnidii Rhizoma, Paeoniae Radix, Ginseng Radix, Poria Sclerotium, Rehmannia Radix, Glycyrrhizae Radix et Rhizoma] (water extract) 678.4 mg/kg, p.o.	Cisplatin (n = 6)	↓	-	↓	Leptin↓ IL-6↓
				Cisplatin + SJDBT (Single) (n = 6)	↑	-	-	Leptin↑ IL-6↑ JAK1/STAT3↑
				Cisplatin + SJDBT (multiple) (n = 6)	↑	-	↑	Leptin↑ IL-6↑
Kim et al. 2017 [47]	SD rats	Single 6 mg/kg, i.p.	Rhus verniciflua Stoke [RVX] (water extract) 25, 50, 100 mg/kg, p.o.	Cisplatin (n = 6)	↓	↑	↓	5-HT↑ 5-HT_3A_ receptor↑ SERT↓ TNF-α↑ IL-6↑ IL-1β↑ WBC↓ Lymphocyte↓ Bone-marrow tissue injury↑ Thymus weight↓ Spleen weight↓
				Cisplatin + RVX (n = 6)	↑	↓	↑	5-HT↓ 5-HT_3A_ receptor↓ SERT↑ TNF-α↓ IL-6↓ IL-1β↓ WBC↑ Lymphocyte↑ Bone-marrow tissue injury↓ Thymus weight↑ Spleen weights↑
Woo et al. 2017 [44]	Balb/c mice	Single 8 mg/kg, i.p.	LCBP-Anocure [LA; Atractylodis Rhizoma Alba (AJ), Angelicae Gigantis Radix (AG), Astragali Radix (AM), Lonicerae Flos (LJ), Taraxaci Herba (TP), Prunellae Spica (PV)]-16001, 16002, 16003 (water extract) 1000 mg/kg, p.o.	Cisplatin (n = 4)	↓	-	↓	Active ghrelin↓ Leptin↓ IL-6↓ p-JAK1↓p-STAT3↓ WBC↓ Neutrophil↓
				Cisplatin + LA16001 (n = 4)	↑	-	↑	Active ghrelin↑ Leptin↑ IL-6↑ p-JAK1↑ p-STAT3↑ WBC↑ Neutrophil↑
				Cisplatin + LA16002 or LA16003 or SJDBT or AJ or AG or LJ or PV (n = 4)	↑	-	-	-
				Cisplatin + MGA (100 mg/kg, p.o.) or AM or TP (n = 4)	↑	-	↑	-
Song et al. 2017 [48]	Wistar rats	Single 5 mg/kg, i.p.	He-Wei granules [HWKL; Pinelliae Tuber, Zingiberis Rhizoma Recens, Ginseng Radix et Rhizoma, Scutellariae Radix, Coptidis Rhizoma, Glycyrrhizae Radix et Rhizoma, Jujubae Fructus] (water extract) 1.18 (low), 2.36 (middle), 4.725 (high) mg/kg, i.g.	Cisplatin (n = 12)	↓	↑	↓	5-HIAA↓ SP↑ 5-HT↑ 5-HIAA/5-HT↓ SERT↓ MAO-A↓ 5-HT_3_AR↑ PPTA↑ TPH-1↑ TPH-2↑ NK-1R↑ TPH-1↑ OB↑ GH↓ GH/OB↓ GPR39↑ EGFR↓ pERK1/2↓ GSH-PX↓ Stomach injury↑ SOD↓ MDA↑ Ileum injury↑
				Cisplatin + HWKL (n = 12)	↑	↓	↑	5-HIAA↑ SP↓ 5-HT↓ 5-HIAA/5-HT↑ SERT↑ MAO-A↑ 5-HT_3_AR↓ PPTA↓ TPH-1↓ TPH-2↓ NK-1R↓ TPH-1↓ OB↓ GH↑ GH/OB↑ GPR39↓ EGFR↑ pERK1/2↑ GSH-PX↑ Stomach injury↓ SOD↑ MDA↓ Ileum injury↓
Kim et al. 2019 [78]	C57BL/6 mice	Multiple (3 times— D1, 6, and 11) 5 mg/kg, i.p.	HemoHIM [Atractylodis Rhizoma Alba, Angelica Gigas Nakai, Astragali Radix] (water extract) 100, 250, 500 mg/kg, p.o.	Cisplatin (n = 7)	-	-	↓	NK cell activity↓ Macrophage phagocytotic activity↓CD4^+^ T lymphocytes↓ Splenocytes↓ IL-2↓ IFN-γ↓
				Cisplatin + HemoHIM (n = 7)	-	-	↑	NK cell activity↑ Macrophage phagocytotic activity↑CD4^+^ T lymphocytes↑ Splenocytes↑ IL-2↑ IFN-γ↑
Chen et al. 2019 [79]	C57BL/6 mice	Multiple (9 times— 3 days/week) 5 mg/kg, i.p.	Zhen-Qi Sijunzi [ZQ-SJZ; Ginseng Radix, Atractylodis Rhizoma Alba, Poria Sclerotium, Glycyrrhizae Preparata, Hedysari Radix, Fructus Ligustri Lucidi] (water extract) 700 mg/kg, p.o.	Cisplatin (n = 18)	↓	-	↓	Intestinal mucosal damage↑
				Cisplatin + ZQ-SJZ (n = 18)	↑	-	↑	Intestinal mucosal damage↓
Goswami et al. 2019 [80]	C57BL/6 mice	Multiple (D0 and 4) 8 mg/kg, i.p.	Ninjin-yoeito [NYT; Angelicae Acutilobae Radix, Atractylodis Rhizoma Alba, Rehmanniae Radix, Poriae Cutis, Ginseng Radix, Citri Unshius Pericarpium Immaturus, Polygalae Radix, Paeoniae Radix, Astragali Radix, Schisandrae Fructus, Glycyrrhizae Radix et Rhizoma] (water extract) 1 g/kg, p.o.	Cisplatin (n = 6)	↓	-	↓	-
				Cisplatin + NYT (n = 6)	↑	-	↑	IR-positive NPY neurons [Ca^2+^]_i_ ↑ Ghrelin-responsive and unresponsive NPY neurons [Ca^2+^]_i_ ↑

Abbreviations: 5-HIAA, 5-Hydroxyindole acetic acid; 5-HT, 5-Hydroxytryptamine (serotonin); EGFR, epidermal growth factor receptor; GH, ghrelin; GHS-R1a, growth hormone secretagogue receptor type 1a; GPR39, G-protein-coupled receptor 39; GSH-PX, glutathione peroxidase; HMF, 3,3′,4′,5,6,7,8-heptamethoxyflavone; IL, interleukin; IFN-γ, interferon-γ; IR, immunoreactive; *Ki*, inhibition constant; MAO-A, monoamine oxidase A; MDA, malonaldehyde; NK cell, natural killer cell; NPY, neuropeptide Y; OB, obestatin; RKT, Rikkunshito; RVX, Rhus verniciflua stoke; SbE, Scutellaria baicalensis extract; SERT, serotonin reuptake transporter; SOD, superoxide dismutase; TNF-α, tumor necrosis factor-α; TPH, tryptophan hydroxylase; WBC, white blood cells.

**Table 2:** Underlying Mechanisms of action of herbal medicine extracts in cisplatin-induced anorexia.

Pathways		Cisplatin	Herbal Medicines		Measured Locations
5-HT	5-HT and 5-HT_3A_ receptor	↑	RVX [47]	↓	Small intestine
			HWKL [48]		Ileum, medulla oblongata, serum
	TPH1		HWKL [57]		Ileum
	TPH2				Medulla oblongata
	SERT	↓	RVX [47]	**↑**	Small intestine
			HWKL [48]		Medulla oblongata, ileum
	5 HIAA				Ileum, serum
Cytokine	IL-6	↓	SJDBT [43], LA16001 [44]	**↑**	Fat, serum
		↑	RVX [47]	↓	Stomach
	IL-1β				
	TNF-α				
		↓	HemoHIM [78]	**↑**	Spleen
	IL-2				
	IFN-γ				
	IL-4	↑		↓	
WBC	Total Number	↑	KG [77]	↓	Serum
		↓	RVX [47], LA16001 [44]	↑	
	Lymphocytes	↑	KG [77]	↓	
		↓	RVX [47]	↑	
			HemoHIM [78]		Spleen
	Neutrophils	↑	KG [77]	↓	Serum
		↓	LA16001 [44]	↑	
	NK cell activity	↓	HemoHIM [78]	↑	
	Macrophage Phagocytotic activity				Peritoneal cavity
	Splenocyte proliferation				Spleen
Hormone	Ghrelin	↓	RKT [45], HWKL [48]	↑	Serum
			LA16001 [44]		Stomach
			HWKL [48]		Antrum
	GHS-R1a		RKT [45]		Hypothalamus
	Leptin		SJDBT [43]		Fat, serum
			LA16001 [44]		Fat, hypothalamus

Abbreviations: ↑, increase; ↓, decrease.

Woo et al. [43] identified that 14 consecutive days of oral administration of SJDBT, an herbal extract mixture consisting of 10 herbal medicines, significantly increased plasma IL-6 levels, which had been decreased by cisplatin. Single SJDBT administration also markedly increased the levels of IL-6 in fat and serum. The authors reported that Janus kinase 1 (JAK1) and signal transducer and activator of transcription 3 (STAT3) mediate the signaling pathway to produce IL-6. Similarly, in another study conducted by Woo et al. [44], LA16001 significantly increased IL-6 levels both in fat and serum, although cisplatin only lowered the IL-6 level in the serum but not in the fat tissue of mice. LA16001 is an herbal-mixture extract composed of six medicinal herbs, with three herbal medicines (Atractylodis Rhizoma Alba, Angelica Gigas Nakai, and Astragali Radix) present in both SJDBT and LA16001. As SJDBT, LA16001 also affected JAK1 and STAT3 pathways, inducing phosphorylation of JAK1 and STAT3 and increasing IL-6.

However, in contrast to the results obtained by Woo et al., Kim et al. [47] reported that IL-6 increased after cisplatin injection, and attenuation of anorexia is related to a decrease in IL-6 in the small intestine of rats. In their study, oral administration of water extract of RVX significantly attenuated abnormal elevations of IL-6 in the stomach, along with other proinflammatory cytokines, such as TNF-α and IL-1β, following cisplatin administration. 

In a study by Kim et al. [78], multiple cisplatin injections significantly decreased the secretion of interferon-γ (IFN-γ) and interleukin-2 (IL-2) and increased interleukin-4 (IL-4) levels in splenocytes obtained from cisplatin-treated mice. Oral administration of HemoHIM, a hot-water extract of Angelica Gigas Nakai, Cnidii Rhizoma, and Paeoniae Radix, upregulated IFN-γ and IL-2 and downregulated IL-4 levels in splenocytes in a dose-dependent manner. In their study, the change in the level of TNF-α after cisplatin injection was not significantly different from that of the control. The authors further assessed that HemoHIM could restore splenocyte proliferation to the control level. Given that IFN-γ and IL-2 are Th1-associated cytokines, whereas IL-4 is a Th2-associated cytokine, the authors assessed that HemoHIM could modulate Th1-/Th2-mediated immune responses in cisplatin-treated mice. 

### 2.3. White Blood Cells (WBCs)

Due to malnutrition, hematological complications are often observed in patients with anorexia [81]. Changes in the total number of WBCs characterized by lymphocytopenia or neutropenia are often present [81,82]. However, the changes in hematological parameters in cisplatin-induced anorexia have not yet been clearly elucidated. Four experiments analyzed the total number of WBCs, neutrophils, lymphocytes, and monocytes after injection of cisplatin and herbal medicines [44,47,49,50]. 

Raghavendran et al. [77] showed that 72 h after intraperitoneal injection of cisplatin (7 mg/kg), the number of WBCs, such as neutrophils, lymphocytes, and monocytes, significantly increased compared to the control. However, oral administration of 50 mg/kg of Korean ginseng root extract (KG) 1 h before cisplatin treatment prevented the increase in hematological parameters. Nevertheless, when KG was treated 2 h after injection of 6 mg/kg of cisplatin, its modulatory effects disappeared. 

In contrast to the study by Raghavendran et al., Woo et al. [44] reported that the number of WBCs and neutrophil decreased when measured 3 days after the injection of 8 mg/kg of cisplatin in mice. LA16001 was administered for three consecutive days, and the number of WBCs and neutrophils in the blood significantly increased. In accordance with the study of Woo et al., Kim et al. [47] reported that 6 mg/kg of cisplatin lowered the number of WBCs and lymphocytes. RVX (100 mg/kg) significantly increased the number of WBCs and lymphocytes. However, the number of neutrophils remained unchanged after cisplatin injection.

In a study by Kim et al. [78], changes in the ratio of CD4^+^/CD8^+^ T lymphocytes, activities of natural killer (NK) cells, and phagocytosis activity of macrophages were assessed. The ratio of CD4^+^/CD8^+^ T lymphocytes and the number of CD4^+^ T lymphocytes are known to determine immunological ability [83]. Although the ratio of CD4^+^/CD8^+^ T lymphocytes was not affected by injection with either cisplatin or herbal medicine, CD4^+^ T lymphocytes significantly increased in splenocytes after medium and high doses of HemoHIM oral treatment. These changes were not observed in the blood. Furthermore, the suppressed activity of NK cells was alleviated after HemoHIM treatment at the highest dose. Moreover, the phagocytic activity of macrophages that initiate the innate immune response decreased significantly following cisplatin injection, whereas HemoHIM treatment significantly alleviated the phagocytic activities of macrophages. Altogether, these results suggest that HemoHIM could restore and enhance immune-cell activity suppressed by chemotherapy treatment.

### 2.4. Ghrelin

Ghrelin is an endogenous ligand of the growth hormone secretagogue receptor 1 (GHS-R1), which consists of 28 amino acids [84]. It is mainly secreted from the stomach, and upon secretion, it increases appetite [85,86,87]. Two major forms of ghrelin are found in the stomach and plasma: acylated ghrelin and desacylated ghrelin [88]. Acylated ghrelin is involved in the regulation of food intake, gastrointestinal motility, and energy expenditure, whereas desacylated ghrelin, although controversial, has been reported to be devoid of the biological activities of acylated ghrelin [84]. When ghrelin is administered peripherally or intracerebroventricularly (ICV) in rodents, it improves gastrointestinal motility and food intake [89,90,91]. In contrast, ICV injection of [D-Lys-3]-GHRP-6, a GHS-R1 antagonist, inhibited food intake increased by ghrelin [59]. Changes in plasma ghrelin levels have been reported in functional dyspepsia, chronic gastritis, and gastric ulcers, suggesting that ghrelin levels are closely associated with gastrointestinal disorders [92,93]. In total, five studies analyzed the modulatory effects of herbal medicines on ghrelin levels [44,45,46,48,80]. 

First, Takeda et al. [45] showed that both plasma-acylated- and -desacylated-ghrelin levels significantly decreased after cisplatin treatment. They also demonstrated that 5-HT_2B_ and 5-HT_2C_ agonists could decrease plasma-acylated-ghrelin levels in rats. Moreover, by demonstrating that cisplatin could significantly decrease acylated-ghrelin levels in vagotomized rats, the authors reported that the action of cisplatin primarily occurs in the peripheral tissues. As mentioned in the 5-HT section, RKT oral treatment succeeded in preventing a cisplatin-induced decrease in plasma-acylated-ghrelin levels when measured 2 h after its administration. They also identified that HMF, hesperidin, and isoliquiritigenin, which are components of RKT, significantly inhibited the cisplatin-induced decrease in plasma-acylated-ghrelin levels. 

In a study by Yakabi et al. [46], cisplatin significantly inhibited relative mRNA levels of GHS-R1a in the hypothalamus but not in the stomach 6 h after the treatment. However, when ghrelin was injected (ICV) into cisplatin-injected rats, no significant changes in food intake were observed compared to saline-treated rats, owing to decreased GHS-R1a in the hypothalamus.

Both Woo et al. [44] and Song et al. [48] showed that herbal medicines could increase ghrelin content in the stomach. Woo et al. showed that oral administration of LA16001 significantly increased acylated-ghrelin levels in the stomach and food intake in cisplatin-injected mice. Song et al. [48] also confirmed the effect of HWKL, as it significantly increased the ghrelin content and the ratio of ghrelin/obestatin in the serum and stomach. 

Goswami et al. [80] demonstrated that oral treatment with 1 mg/kg/day of ninjin’yoeito (NYT) for 10 days could increase both food intake and body weight in cisplatin-treated mice. To further analyze the mechanism of action of NYT, they focused on first-order neurons of the arcuate nucleus (ARC), which are known to receive feeding-related signals from the periphery [94]. NYT (10 μg/mL) administration for 10 min increased cytosolic Ca^2+^ concentration ([Ca^2+^]_i_) in a single neuron isolated from ARC, showing that NYT could modulate the action of ARC neurons. Furthermore, both NYT and ghrelin (10^−8^ M) increased [Ca^2+^]_i_ in ARC neurons immunoreactive to neuropeptide Y (NPY-IR). NPY neurons are reported to play a critical role in feeding regulation [95], as their deletion results in reduced feeding and body weight in adult mice [96,97]. In addition, NYT increased [Ca^2+^]_i_ in ghrelin-responsive and -unresponsive NPY neurons. Among 81 single ARC NPY-IR neurons, eight neurons (9.9%) responded to NYT, only 30 (37.0%) responded to ghrelin only, 18 (22.2%) responded to both, and 25 (30.9%) responded to none. Among 26 NPY neurons that responded to NYT, 18 (69.2%) responded to ghrelin, indicating that NYT activates both ghrelin-responsive (69.2%) and ghrelin-unresponsive (30.8%) NPY neurons.

### 2.5. Leptin

Leptin is a hormone secreted by fat cells that is known to reduce body fat and increase energy expenditure. Since its discovery in 1994, leptin has gained much attention in feeding regulations [98]. Although its role in chemotherapy-induced anorexia remains unclear, it is seen as an indicator of the severity of the disorder, as serum leptin levels have been reported to be low in anorexia-induced humans and rodents [16,99]. 

In a study by Woo et al. [43], 8 mg/kg of cisplatin significantly reduced serum and fat leptin levels in mice. However, multiple SJDBT administration for 14 days, starting from three days after cisplatin injection, significantly increased both serum and fat leptin levels. Single SJDBT administration also succeeded in ameliorating leptin levels in fat and serum. The authors further suggested that SJDBT administration could increase leptin by activating the JAK1/STAT3-mediated signaling pathway in adipocytes, as demonstrated by Western blotting in fat tissues. Furthermore, in their next study, Woo et al. [44] showed that cisplatin injection reduced leptin in fat but not in the hypothalamus 4 h after its injection in mice. Although leptin levels were not altered in the hypothalamus, LA16001 significantly increased leptin levels in adipocytes. 

### 2.6. Most Used Herbal Medicines

Ginseng Radix was mentioned in seven studies [43,45,46,48,50,80,81], and both Glycyrrhizae Radix et Rhizoma [45,46,48,80] and Angelicae Gigantis Radix [43,44,49,81] were included in four herbal medicine extracts. Pinelliae Tuber [45,46,48], Zingiberis Rhizoma [45,46,48], Astragali Radix [43,44,81], and Paeoniae Radix [43,49,81] were used in three studies each. Scutellariae Radix [48,75], Cinnamomi Cortex [43,80], and Rehmanniae Radix [43,80] were only mentioned in two studies each.

## 3. Discussion

In this review, we examined the effects of herbal medicines in an animal model of cisplatin-induced anorexia. A total of 12 papers were included. Eight studies used a mixture of various herbal medicine extracts [43,44,45,46,48,78,79,80], and four used single herbal medicine extracts [47,50,78,79]. To our knowledge, this is the first review to summarize and analyze the effects of herbal medicines in an animal model of cisplatin-induced feeding disorder.

With the increase in the number of cancer patients, cisplatin has been cited as one of the most used anticancer medications due to its broad efficacy in the treatment of cancers [100]. However, anorexia is a serious dose-limiting side effect caused by cisplatin that could decrease survival [101], as well as quality of life in cancer patients [102]. Thus, it is critical to find new approaches for drug development, as the number of drug options is limited. In the included studies, cisplatin was administered to rodents to mimic feeding disorders induced in clinical settings. Most studies used a single cisplatin injection; however, in two studies, cisplatin was injected multiple times (3 and 15 times) [78,79]. In addition, the doses used were also different for each experiment, with the lowest dose being 2 mg/kg [45,46] and the highest dose being 8 mg/kg [43,44,81] (Table 1). The therapeutic range for cisplatin in humans (60 kg) is 35 mg/m^2^, which corresponds to approximately 5 mg/kg in rodents [103]. 

**Figure 1:**
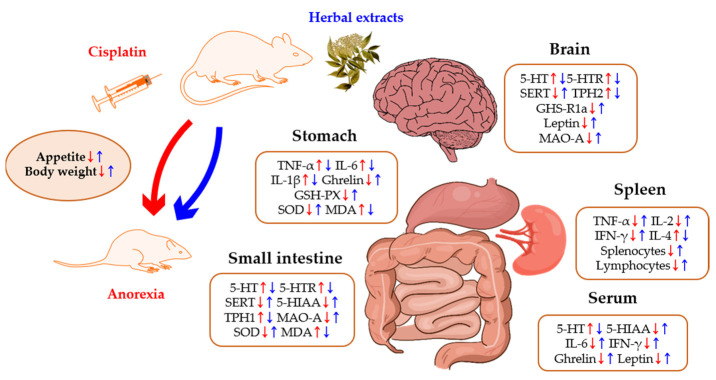
The pathogenesis mechanism of cisplatin-induced anorexia and the mechanisms of action of herbal extracts. Intraperitoneal administration of cisplatin induces anorexia (**red**), whereas administration herbal extract increases appetite (**blue**). The brain, serum, small intestine, stomach, and spleen were affected by both cisplatin and herbal-extract treatments. Abbreviation: GHS-R1a, growth hormone secretagogue receptor type 1a; GSH-PX, glutathione peroxidase; 5-HIAA, 5-Hydroxyindole acetic acid; HMF, 3,3′,4′,5,6,7,8-heptamethoxyflavone; 5-HT, 5-Hydroxytryptamine (serotonin); 5-HTR, 5-HT receptor; IFN-γ, interferon-γ; MAO-A, monoamine oxidase A; MDA, malonaldehyde; SERT, serotonin reuptake transporter; SOD, superoxide dismutase; TNF-α, tumor necrosis factor-α; TPH, tryptophan hydroxylase.

As for the pathogenesis mechanism of anorexia, changes in 5-HT, inflammatory cytokines, WBCs, ghrelin, and leptin were analyzed in the studies (Table 2 and Figure 1). 

When the level of 5-HT is increased, it is known to induce a feeling of satiety and reduced appetite [104,105,106]. There are reports confirming that plasma 5-HT levels are elevated in cancer patients [107,108,109] and in the small intestine of animals [110,111] after cisplatin treatment. In accordance with these results, in the included studies, 5-HT was shown to be increased after cisplatin injection in the small intestine, serum, and brain, whereas the administration of herbal medicine (RVX and HWKL) succeeded in reducing 5-HT levels and ameliorating food intake in mice [47,48]. Along with 5-HT, 5-HT receptors are also closely involved in anorexia. Takeda et al. [45] and Yakabi et al. [46] suggested that the activation of 5-HT_2B_ and 5-HT_2C_ receptors may induce anorexia, as injection of their antagonist prevented a decrease in food intake. The activities of 5-HT_2B_ and 5-HT_2C_ receptors have also been related to loss of appetite and decreased food intake in other studies [112,113,114]. Although 5-HT_2B_ receptors are mostly peripherally distributed, such as in the gastrointestinal tract, stomach fundus, and blood vessels, 5-HT_2C_ receptors are known to exist centrally, mainly in the brain. 5-HT_2C_ receptor stimulation by administration of selective agonists is known to induce a marked decrease in food intake, and knockout of the 5-HT_2C_ receptor subtype in mice caused leptin-independent hyperphagia and hypoactivity, leading to obesity [115]. Contrary to 5-HT_2B_ and 5-HT_2C_ receptors, the role of 5-HT_3_ receptors in anorexia is still controversial. 5-HT_3_ receptors have been considered to play an important role in GI tract diseases, and antagonists such as ondansetron have been used for the prevention and treatment of vomiting [116,117]. However, in the included studies, subcutaneous administration of ondansetron, a 5-HT_3_ receptor antagonist, had no effect on the decrease in plasma-ghrelin concentration and food intake caused by cisplatin, suggesting that 5-HT_3_ receptors may not be closely involved in anorexia [45]. Furthermore, although Kim et al. [47] and Song et al. [48] showed a change in 5-HT_3A_ receptor mRNA expression in the mouse brain and small intestine after cisplatin treatment, ondansetron failed to increase food consumption, suggesting that modulating the activity of 5-HT_3A_ receptors may not be effective for the treatment of anorexia.

Regarding the change in inflammatory cytokines after cisplatin injection, TNF-α, IL-1β, and IL-6 increased in the stomach tissue when measured 5 days after a dose of 6 mg/kg of cisplatin [47]. In splenocytes, IL-2 and IFN-γ levels decreased, while IL-4 levels increased. TNF-α levels remained unchanged 14 days after multiple cisplatin injections [78]. In the studies by Woo et al. [43,44], IL-6 slightly decreased only in the serum when measured 4 h after 8 mg/kg of cisplatin treatment but not when measured 14 days after cisplatin injection in the serum or fat. Among the treatments, RVX significantly attenuated the increased TNF-α, IL-1β, and IL-6 levels in the stomach [47], and HemoHIM increased IL-2 and IFN-γ levels [78]. Both SJDBT [43] and LA16001 [44] significantly increased IL-6 levels in serum and fat tissue. RVX, SJDBT, and LA16001 all succeeded in decreasing anorexia in rodents, but their effects on IL-6 were different. The pathogenesis of inflammation-associated anorexia induced by cisplatin is unclear, and the role of IL-6 needs further investigation, as IL-6 was also shown to be highly elevated in both anorexia and obese patients [118].

The total WBC count was also different, according to previous studies. WBC decreased after cisplatin injection and increased after herbal medicines in two studies [44,47], whereas in the study by Raghavendran et al. [77] WBC increased after cisplatin treatment and decreased after KG administration. The difference in the WBC count (k/μL) after cisplatin injection may be due to the difference in the measurement time (48 h [44], 72 h [77], or 120 h [47]), animals used (Balb/c mice [44] or SD rats [78]), or the dose used (6 mg/kg [47], 7 mg/kg [77], or 8 mg/kg [44]).

The most frequently used herb in the 12 papers was Ginseng Radix, which was included in six herbal mixtures [43,45,46,50,80,81]. The components contributing to the therapeutic effect of ginseng radix on various diseases have been well studied, and it has been proven that Ginseng Radix has anticancer, antioxidant, and anti-inflammatory effects [119,120,121]. In particular, the anti-inflammatory effect of Ginseng Radix may have been effective in treating anorexia. For example, it has been reported that ginseng, a polysaccharide extracted from Ginseng Radix, inhibits the p38 MAP kinase pathway and NF-*κ*B in vitro and inhibits proinflammatory cytokines in vivo [122]. Ginsenoside Rg3, another component of Ginseng Radix, was shown to inhibit phorbol-ester-induced COX-2 and NF-*κ*B induction [123].

Angelicae Gigantis Radix was also included in four studies [43,44,49,81], making it the second most used medicinal herb among the reviewed studies. Angelicae Gigantis Radix attenuated scratching behavior, and 2,4-dinitrochlorobenzene-induced atopic dermatitis-like skin lesions by reducing serum IgE, histamine, TNF-α, IL-6, and COX-2 expression in skin tissue from mouse models [124]. Atractylodis Rhizoma Alba is a medicinal herb that was used in three of the papers reviewed [43,44,81]. Atractylon, which is one of the components of Atractylodis Rhizoma Alba, significantly inhibited NO and prostaglandin E2 production, as well as inducible NO synthase and cyclooxygenase-2 expression, in LPS-induced RAW 264.7 cells [125].

For future studies, it would be valuable to observe the effect of herbal-mixture extracts that have Ginseng Radix, Angelicae Gigantis Radix, or Atractylodis Rhizoma as their components. As a candidate, Bojungikki-tang could be considered, as it is a mixture of eight herbal extracts, including Ginseng Radix, Angelicae Gigantis Radix, and Atractylodis Rhizoma. Moreover, it has been widely used in the past to improve problems associated with digestive disease, such as decreased gastrointestinal motility and gastric injury [126,127]. In addition, ban-xia-xie-xin-tang and dai-kenchu-to could also be effective against cisplatin-induced anorexia, as they both have Ginseng Radix as a component. Ban-xia-xie-xin-tang is composed of seven herbs and has been reported to be effective for the treatment of various digestive inflammations, such as colitis, esophagitis, and gastritis [39,128]. In addition, dai-kenchu-to has been reported to increase blood flow in the intestinal tract [129], stimulate intestinal motility [130,131], and prevent bacterial translocation [132]. 

In conclusion, based on the results obtained from all included studies, we suggest that herbal medicines could be considered as an effective treatment method for cisplatin-induced anorexia. However, more well-designed clinical trials and experimental studies should be conducted to clarify their effect and to increase the understanding of the mechanisms of action. We believe that this review will help other researchers in the field of herbal medicines and cisplatin-induced anorexia to better understand its mechanism in the future. 

## 4. Methods

**Figure 2:**
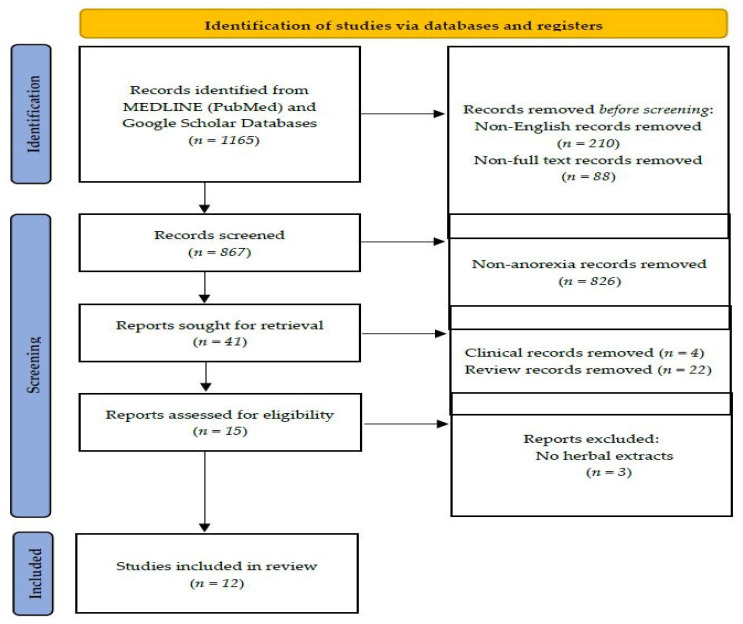
Flow chart of article inclusion protocol. Identification through searches of MEDLINE (PubMed) and Google Scholar yielded 1165 articles, which were screened by abstract and full-text examinations. Finally, a total of 12 articles assessing the effect of herbal extracts in cisplatin-induced anorexia in rodents were included in our review.

A search was conducted of all studies on herbal medicines and cisplatin-induced anorexia in the National Library of Medicine (MEDLINE) using PubMed, and Google Scholar (Figure 2). Extensive searches were undertaken for articles written in English, as non-English studies were excluded. Studies electronically published until the end of September 2021 were included. The literature search was performed using the following keywords: “anorexia”, “cisplatin”, “feeding disorder”, and “herbal medicines”. After the initial search, duplicates, bibliographies, study protocols, clinical trials, and non-English studies were excluded. Twelve animal studies were included in this study.

## Data Availability

Not applicable.
